# Detection of Lower Body for AGV Based on SSD Algorithm with ResNet

**DOI:** 10.3390/s22052008

**Published:** 2022-03-04

**Authors:** Xinbiao Gao, Junhua Xu, Chuan Luo, Jun Zhou, Panling Huang, Jianxin Deng

**Affiliations:** 1 School of Mechanical Engineering, Shandong University, Jinan 250061, China; gxb@mail.sdu.edu.cn (X.G.); 202134374@mail.sdu.edu.cn (J.X.); 201933994@mail.sdu.edu.cn (C.L.); hfpl@sdu.edu.cn (P.H.); jxdeng@sdu.edu.cn (J.D.); 2 Key Laboratory of High Efficiency and Clean Mechanical Manufacture, School of Mechanical Engineering, Shandong University, Ministry of Education, Jinan 250061, China; 3 Shandong Alesmart Intelligent Technology Co., Ltd., Jinan 250061, China; 4 National Experimental Teaching Demonstration Center of Mechanical Engineering, School of Mechanical Engineering, Shandong University, Jinan 250061, China; 5 Shandong Institute of Industrial Technology, Jinan 250061, China

**Keywords:** object detection, SSD, ResNet

## Abstract

Detection of human lower body provides an implementation idea for the automatic tracking and accurate relocation of automatic vehicles. Based on traditional SSD and ResNet, this paper proposes an improved detection algorithm R-SSD for human lower body detection, which utilizes ResNet50 instead of VGG16 to improve the feature extraction level of the model. According to the application of acquisition equipment, the model input resolution is increased to 448 × 448 and the model detection range is expanded. Six feature maps of the updated resolution network are selected for detection and the lower body image dataset is clustered into five categories for aspect ratio, which are evenly distributed to each feature detection map. The experimental results show that the model R-SSD detection accuracy after training reaches 85.1% mAP. Compared with the original SSD, the detection accuracy is improved by 7% mAP. The detection confidence in practical application reaches more than 99%, which lays the foundation for subsequent tracking and relocation for automatic vehicles.

## 1. Introduction

As an important carrier of logistics and transportation, automatic guided vehicles are developing rapidly. Various intelligent factories have introduced intelligent transportation systems to improve production efficiency. However, the application scenarios of the industrial workshop are more and more complex, so the requirements for the accuracy, stability and flexibility of AGV are also increasing. With the development of computer technology and artificial intelligence, vision technology has been paid great attention in the field of research and production. Effective detection and tracking in the process of intelligent factories has great application prospects. Due to the low base of the automatic guided vehicle, the perceptual visual range is mainly concentrated in the area above the ground. In order to ensure the accuracy of detection, the human lower body is selected as the object to be detected. Therefore, it is particularly important to propose a detection method suitable for the human lower body.

In recent years, deep learning, especially convolutional neural networks, has performed very well in the field of object detection. The popular detection methods can be divided into two categories: a two-stage object detection algorithm and a one-stage object detection algorithm. The representative of two-stage target detection is Faster R-CNN [1], and the representatives of one-stage target detection are YOLO [2] and Single Shot Multibox Detector (SSD) [3]. Among them, SSD shows a good balance between speed and accuracy.

In the engineering application, Kumar et al. [4] demonstrated an approach to train CNN based on SSD and MobileNet, which improved detection speed and accuracy. Biswas et al. [5] implemented SSD to estimate traffic density. Gupta et al. [6] analyzed the two algorithms YOLOv3 and SSD to count the number of people at any junction. Their results have shown that SSD is better than YOLOV3 v3. Zimoch et al. [7] proposed a centroid algorithm combined with the Siamese network based on the pretrained SSD method in people detection. Their approach involved on-edge image analysis to decrease the risk of data loss and the system cost. Ahmed et al. [8] proposed an SSD model with Mobilenetv2 as the basic network to detect people. The detection model’s accuracy was enhanced with a transfer learning approach. To speed up the computational performance of the people detection technique, Kumar et al. [9] used the SSD algorithm along with the help of the architecture of a faster region convolutional neural network. Jang et al. [10] proposed the Face-SSD method using a Fully Convolutional Neural Network (FCNN) to detect multiple faces of different sizes and recognize one or more face-related classes. Nagrath et al. [11] proposed the SSDMNV2 approach to detect faces using SSD as a face detector and MobilenetV2 architecture as a framework for the classifier.

In terms of feature extraction networks, researchers have developed more and more efficient network structures [12,13,14], such as VGGNet [15], GoogLeNet [16], ResNet [17] and so on. SSD adopted the network structure of VGG16, which changed the full connection layer to the full convolution layer and added several layers for auxiliary feature extraction. Aiming at a low level of small target detection, Zhai et al. [18] proposed an improved algorithm based on the DenseNet backbone network and feature fusion, which improved the performance of the model and had a good detection effect for small objects. Hao et al. [19] fused a deformable convolution network and SSD, which adapted to the geometric changes of image content and effectively improved the accuracy of general object detection.

With the design of the network structure becoming more perfect, the effect is getting better in public datasets, such as Pascal VOC [20] and MS COCO [21]. However, in engineering applications, an appropriate training model should be selected according to the needs of the scene. In terms of appearance, the physical model of the human lower body is relatively simple. In the process of deep-seated network training, it is easy to appear the phenomenon of model overfitting or model degradation. Therefore, this paper selects ResNet to improve the ability of feature expression of the model instead of original network structure VGG. When an object is tracked, the position of the object is relatively fixed in space. The size and aspect ratio of the object are linearly distributed. Therefore, based on the aspect ratio of the human lower body, the size of the original prediction frame of SSD is adjusted to multiple distribution to improve the detection accuracy of the model. This paper is ordered as follows. The methodologies of SSD and ResNet are introduced in Section 2. The implementation principle of R-SSD is described in Section 2.2. Section 3 describes the datasets used for the detection experiment and the experimental results. Discussions of R-SSD are presented in Section 4. Finally, the conclusion is presented in Section 5.

## 2. Materials and Methods

To build a target detection model, it is necessary to design a convolutional neural network learning framework to train the labeled target sample data after target sample production and image target detection preprocessing.

### 2.1. SSD

SSD is a “one-step” feedforward detection framework based on a deep convolutional neural network. The framework generates a set of predictive regression boxes and the confidence score set of objects in these boxes, and then obtains the final result through the non-maximum suppression (NMS) method.

**Figure 1:**
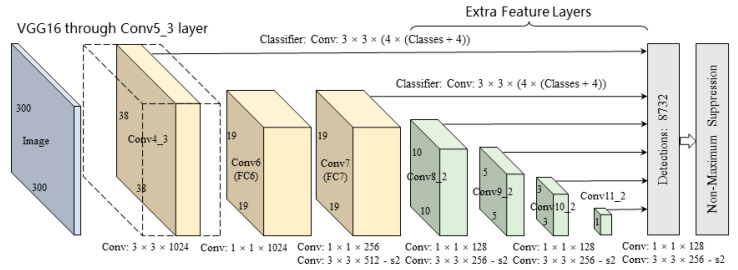
SSD object recognition algorithm architecture.

Figure 1 illustrates the SSD architecture. The framework utilizes the standard network VGG of high-quality image classification as the basic network architecture and modifies the last two layers of the full connection layer into the convolution layer. Extra convolution layers are added for feature extraction with lower resolution. The framework is called a full convolution network, which can adapt to images of various sizes and is no longer subject to the size of the input image.

**Figure 2:**
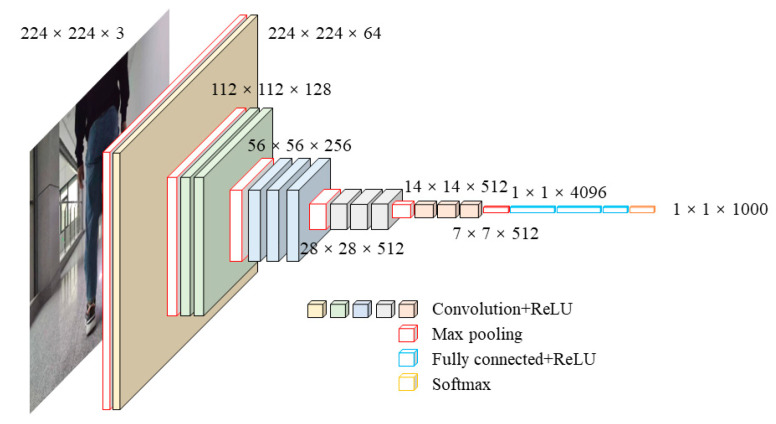
The visualization architecture of VGG network 2.2 The residual network of ResNet.

The architecture of the VGG network is composed of 5 convolutional layers and 3 fully connected layers, and the activation units of all hidden layers adopt the ReLU function. The structure of multiple convolutional layers alternating with nonlinear activation layers performs better than the structure of a single convolutional layer in extracting deeper features. The visualization of the VGG architecture is shown in Figure 2.

The auxiliary structure is added to the network to generate detection with the following key features: multi-scale feature map and multi-aspect ratio bounding regression box. Using VGG as a reference, SSD adopts small convolution filters to predict the class fraction and position offset of a set of default bounding boxes on the feature map, which does not need to resample the bounding box features. In order to achieve high detection accuracy, different scale predictions are produced from different scale feature maps and the prediction is clearly separated by aspect ratio. The low-level feature maps predict small objects and the high-level feature maps predict large objects.

In general, each layer of the neural network corresponds to the extraction of feature information of different levels, including low level, middle level and high level. The deeper the network is, the more information at different levels will be extracted, and the more combinations of information at different levels there will be. The VGG network tries to explore the depth of the deep learning network to continuously improve classification accuracy. However, when CNN reaches a certain depth, the increasing number of layers does not bring further improvement in classification performance but leads to slower network convergence and worse classification accuracy of the test dataset. In view of this, ResNet is proposed to solve the problem of degradation.

**Figure 3:**
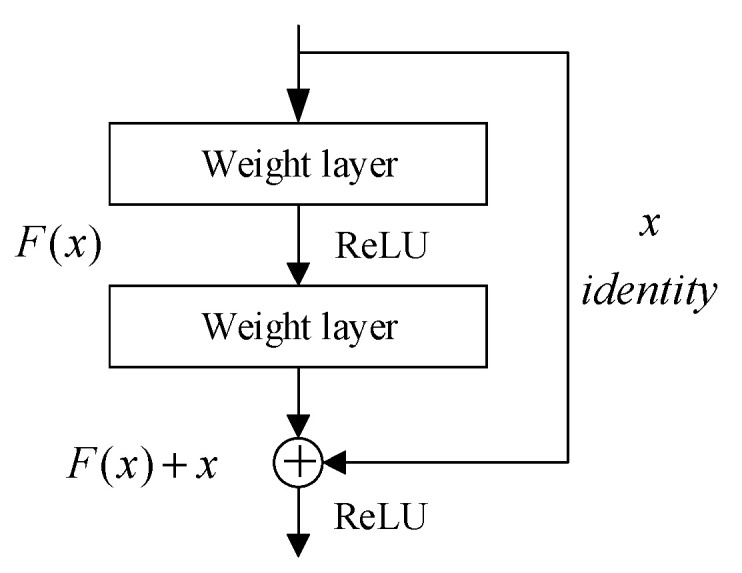
The residual network of ResNet.

ResNet is a residual network module. The residual structure associates input and output channels through “shortcut connections.” It can be understood as a sub-network and can be stacked to form a deep network, which not only ensures that the network can achieve a deep and complex structure and improve the ability of feature expression, but also solves the problems of overfitting and degradation that can easily occur in the network. Assuming that the expected output of the underlying mapping is H(x) and that residual feature mapping is F(x)=H(x)−x, it is easier to optimize the residual to 0 than to optimize the original underlying mapping. Figure 3 shows the residual network.

### 2.2. Improved SSD with ResNet (R-SSD)

**Figure 4:**
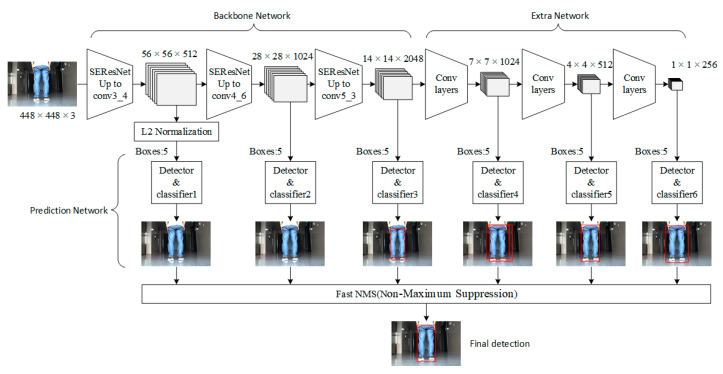
The structure of improved SSD with ResNet (R-SSD). In the figure, the backbone network is used for feature extraction; the extra network is used to extract deeper features and the prediction network is used to detect objects on a multi-scale feature map, including category prediction and position regression.

The network structure of improved SSD with ResNet (R-SSD) can be divided into three parts: the backbone network ResNet for feature extraction, the extra network for extracting deeper features and the prediction network for object detection on a multi-scale feature map, including category prediction and position regression. The structure of the whole model is shown in Figure 4.

#### 2.2.1. Model Input Resolution

The image input resolution of the model is 448 × 448 in order to adapt to the resolution of the image acquisition camera, and different size feature maps are obtained through a classification network. Compared with the original 300 × 300, the feature of the object can be extracted better from a larger image range.

#### 2.2.2. Feature Extraction Layer

**Figure 5:**
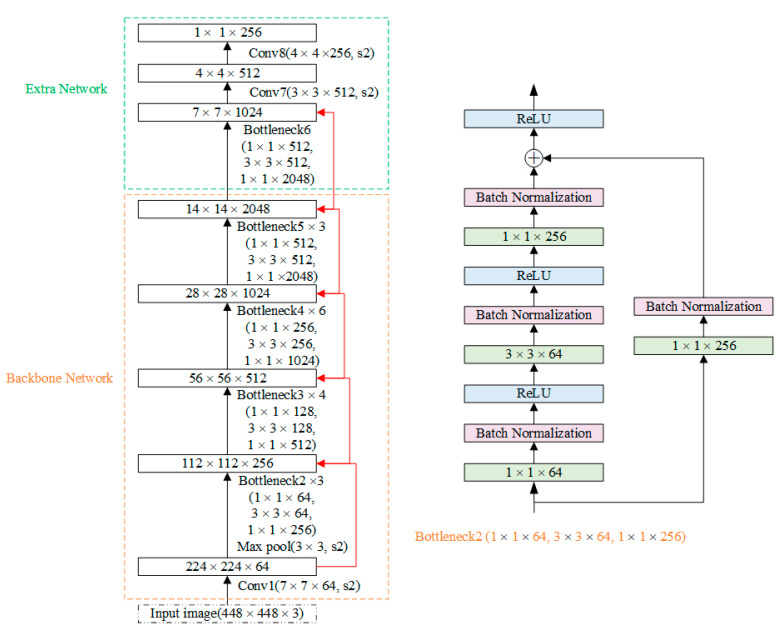
The structure of the feature extraction network of R-SSD.

Because it is easy to change dimensions, design network flexibility, and reduce the amount of computing, we choose the convolution layer modules (called “Bottleneck”) in ResNet50 to replace the VGG16 network of the original SSD. In order to ensure the accuracy of detection, all the feature maps in R-SSD are processed by Batch Normalization and nonlinear activation function ReLU to improve the feature expression ability of network structure and to avoid the overfitting phenomenon. To better preserve the expression ability of features, the convolution kernel with a step size of 2 is used to replace the pooling layer used for feature compression in the original SSD. Figure 5 shows the network structure of the feature extraction.

In Figure 5, the left part shows two parts of the feature extraction network: backbone network and extra network. Taking Bottleneck2 as an example, the right part shows the specific internal structure of each residual block. A “short connection” is realized through a 1 × 1 convolution kernel performing feature channel fusion between each block.

#### 2.2.3. Multi-Scale Object Detection

In the original SSD, Conv4_3, Conv7 of VGG16, and added Conv8_2, Conv9_2, Conv10_2 and Conv11_2 layers are selected for object classification and position regression. We constructed a multi-scale feature extraction layer based on SSD. Due to the difference in input size, the size of the feature extraction layer changes according to the structure of the human lower body and the general position occupied in the image. Bottleneck3_4, Bottleneck4_6, Bottleneck5_3 and added Bottleneck6, Conv7 and Conv8 layers are utilized as object detection layers. The size of the feature layer group is composed of six scales: 56 × 56, 28 × 28, 14 × 14, 7 × 7, 4 × 4, 1 × 1. The number of default boxes in each layer of the feature layer group is distributed according to {5, 5, 5, 5, 5, 5} (see D. Box Clusters) and the total number is 20910, which is far more than that of the original SSD. The specific parameters of the multiscale detection network are shown in Table 1. For the Bottleneck3_4 feature map of R-SSD, since the network layer is in the front and the variance is relatively large, L2 normalization technology is adopted to adjust the feature norm of all outputs to 20.

#### 2.2.4. Bounding Box Clusters

The object position in this study is relatively fixed and the degree of change is relatively small. In order to detect the object location more accurately, we use the dimension clusters in YOLO9000 [22] for reference and cluster the size of the bounding box in the training dataset. The clustering criteria are as follows:

(1) d(box,centriod)=1−IOU(box,centriod)

**Figure 6:**
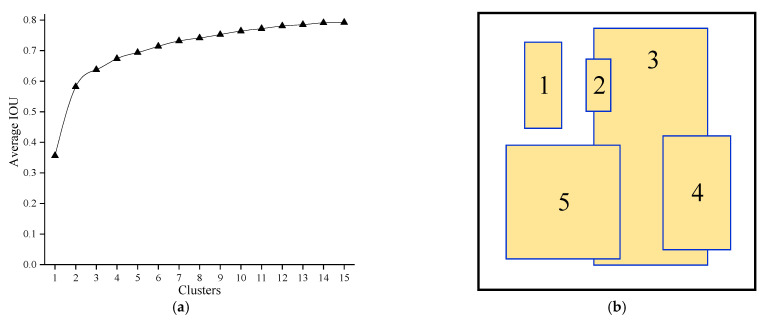
(**a**) The relationship between the number of clusters and the average IOU; (**b**) Dataset clustering bounding boxes.

According to the above criteria, the bounding boxes of the sample data are divided into five categories, which replace the original manual default boxes in the six feature maps. The relative size is shown in Table 2 and the size of the unified dimension is shown in Figure 6. On the premise of ensuring the number of default boxes, we can improve the speed and accuracy of the detection process. The default boxes after clustering are longer and narrower than the original manual calculation, which conforms to the contour of the human lower body.

The R-SSD algorithm comprehensively considers the loss of location and confidence, which follows the original SSD loss calculation criteria. Based on this, the performance of the algorithm is evaluated. The loss function is calculated as follows:

(2) $$L\left(x,c,l,g\right)=\frac{1}{N}\left(L_{conf}\left(x,c\right)+\alpha L_{loc}\left(x,l,g\right)\right)$$

where *N* is the number of default boxes with successful detection. If *N* = 0, the sum of losses is zero. When the cross-entropy loss is used for evaluation, the weight coefficient α is set to 1.

The location loss is a Smooth L1 loss [23] to measure the error between the predicted box (l) and the ground truth box (g) parameters. The bias of the center point of the box (*cx*, *cy*) and the width (*w*) and height (*h*) of the pre-selected box are predicted by regression according to the gradient descent direction of the loss.

(3) $$L_{loc}\left(x,l,g\right)=\overset{N}{\underset{i\in Pos}{\sum }}\underset{m\in \left\{cx,cy,w,h\right\}}{\sum }x_{ij}^{k}smooth_{L1}\left(l_{i}^{m}-{\hat{g}}_{j}^{m}\right)$$

$${\hat{g}}_{j}^{cx}=\frac{\left(g_{j}^{cx}-d_{i}^{cx}\right)}{d_{i}^{w}}$$

$${\hat{g}}_{j}^{cy}=log\frac{\left(g_{j}^{cy}-d_{i}^{cy}\right)}{d_{i}^{h}}$$

$${\hat{g}}_{j}^{w}=log\left(\frac{g_{j}^{w}}{d_{i}^{w}}\right)$$

(4) $${\hat{g}}_{j}^{h}=log\left(\frac{g_{j}^{h}}{d_{i}^{h}}\right)$$

where $x_{ij}^{k}\in \{0,1\}$, it indicates whether the i-th predicted box matches the j-th ground truth box. If the value is 1, the matching correct category is k.

(5) $$smooth_{L1}\left(x\right)=\{\begin{array}{c} 0.5x^{2},\left|x\right|<1\\ \left|x\right|-0.5,otherwise \end{array}$$

The confidence loss is a softmax loss based on multi-classification probability.

(6) $$L_{conf}\left(x,c\right)=-\overset{N}{\underset{i\in Pos}{\sum }}x_{ij}^{p}log\left({\hat{c}}_{i}^{p}\right)-\underset{i\in Neg}{\sum }log\left({\hat{c}}_{i}^{0}\right)$$

where

(7) $${\hat{c}}_{i}^{p}=\frac{exp\left({\hat{c}}_{i}^{p}\right)}{\sum_{p}exp\left({\hat{c}}_{i}^{p}\right)}$$

Implementation of SSD and R-SSD: For an object detection algorithm, creating training datasets is a meticulous process. We marked 1367 objects over 1132 images. All label images create an XML file that contains detailed information about the label object (location, height and width). Before the label dataset is submitted to SSD, a mapping of the dataset location is created. The batch size we used is 32. The base network layers are initialized with the parameters trained on VOC2007, and the extra convolution layers are initialized with the “kaiming” method [24]. Most of our training strategies follow SSD, including loss function, data augmentation and so on. The latest SSD result, including a random data augmentation strategy, has proved to be very useful in detecting small objects and this strategy is also used in the R-SSD framework. The hard negative mining technique of SSD adopted to make the proportion of positive and negative samples is at most 3:1, which makes the optimization faster and training more stable. During the training process, we set the learning rate to 0.0005. After that, the learning rate will gradually decrease according to the number of iterations to obtain more accurate training results. At the end of the training process, a pth file is created for object detection.

#### 2.2.5. Dataset

**Figure 7:**
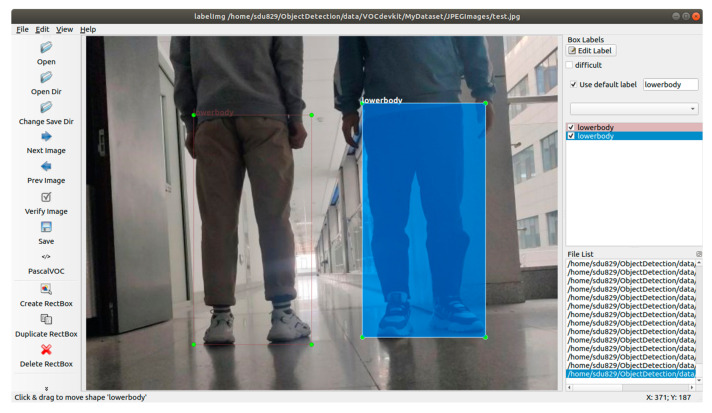
Labeled lower bodies shown in software ‘LabelImg’.

A dataset is an important part of CNN research, especially in the training and validation phase. The quality of the data affects the research effect. The data are collected by Daheng image industrial camera in our work. The pixel size is 640 × 480 pixels, which comes from the factory workshop, laboratory environment and part of the network. The annotation of the image objects is done using the open-source software “LabelImg” [25]. The label process is presented in Figure 7.

In our work, the lower body is labeled as a unified label to obtain more accurate multi-directional feature information because the view of tracking is mainly the back of the human body and the front and back features are highly similar. Given the factory application scenario, training people have distinctive leg shapes, such as overalls and jeans, excluding data with obvious occlusion, such as skirts. Training images with a single background and complete object are added to enhance the training of image data with partial occlusion. Training models (pth) are created simultaneously, as one of the purposes of this study is to understand the influence of background complexity on training accuracy. Table 3 describes the number of complex images and simple images that are tagged to create the same training dataset.

## 3. Results

**Figure 8:**
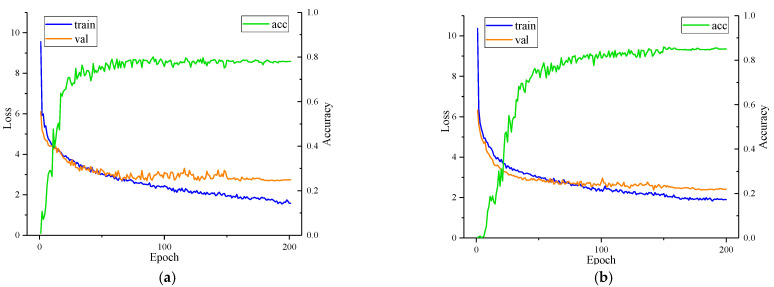
Training process loss curve of SSD based on 300 × 300 pixels and R-SSD based on 448 × 448 pixels. The red line represents the loss of the training process, and the black line represents the loss of the validation process. Because the training data structure is relatively simple and the validation loss is the result of training after an epoch, the validation loss is slightly lower than the training loss in the initial stage. (**a**) SSD training process loss; (**b**) R-SSD training process loss.

SSD and R-SSD are tested on 268 sample images and the accuracy of the algorithm is evaluated by training loss and recognition accuracy. Using NVIDIA GTX 2080Ti GPU to train 200 epochs, the average time of each epoch is about 15 s and the average training time of the system is 50 min. In the process of training, we train and verify at the same time and observe the visualization process of network model changes so as to make the network super parameters better. The loss changes in the training process are shown in Figure 8. Each epoch will create a model and the best one is the model corresponding to the epoch with the lowest loss in the verification phase. The accuracy of the following experimental results comes from the best model, as shown in Table 4.

### 3.1. Training Model

First, we compare the influence of different backbone models on the recognition accuracy of the human lower body. As can be seen from the first and second rows of Table 4, compared with the original SSD (78.1% mAP), SSD based on ResNet (80.7% mAP) has an accuracy improvement of 2.6%.

### 3.2. Input Size

Analyzing rows 2 and 3 in Table 4, when the training input size is 300, the detection accuracy of the model is 80.7% mAP. While the training input size is 448, the detection accuracy of the model is improved to 83.0% mAP. This means that the larger the input size, the better the detection results will be. However, in order to avoid increasing the detection time due to the large input size, we set 448 × 448 as the final model input size combined with the use of the camera resolution.

### 3.3. Data Expansion

Compared with rows 3 and 4 in Table 4, the detection effect is improved significantly with an improvement rate of 1.5% (83.30% mAP vs. 84.5% mAP) after adding training data with a simple background and obvious object characteristics. This shows that the enhancement of the object data sample has an obvious effect and can reduce the information loss of the original input image.

### 3.4. Bounding Box Clustering

From the last two rows of Table 4, it can be seen that the detection accuracy after using bounding box clustering (85.1% mAP) is slightly improved compared with that without bounding box clustering (84.5% mAP). This shows that the method is effective and feasible and the effect will be more significant under the condition of sufficient data.

### 3.5. Visualization

**Figure 9:**
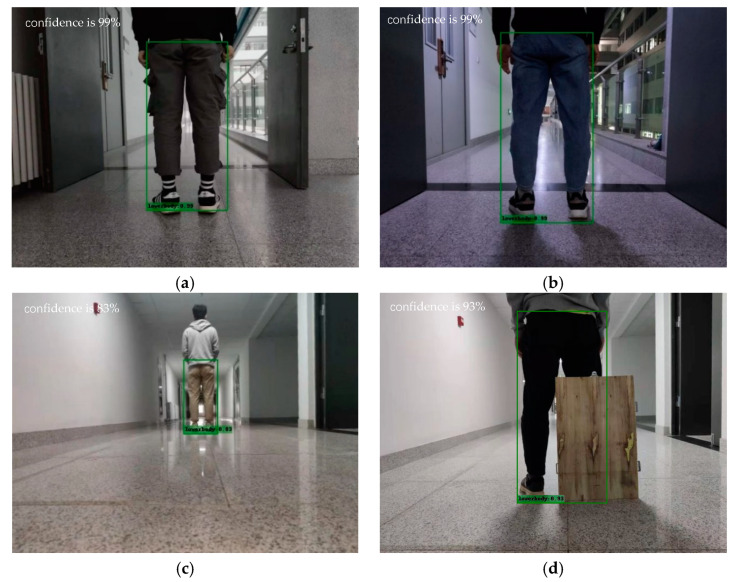
Object detection results in different environments. (**a**) Sufficient illumination; (**b**) Dim illumination; (**c**) Object scale change; (**d**) Object occlusion.

Figure 9 shows the recognition results of the object in different environments. Under the four conditions of illumination, object scale change and occlusion, the lower body can be accurately recognized and detected. The recognition confidence is 99%, 99%, 83%, and 93%, respectively and the average recognition confidence is 93.5%. When the object is far or the object is blocked by obstacles, the detection accuracy will be lower if the camera feels less objects in its’ field of vision. When the object is close, the camera feels more objects in its’ field of vision, and the detection accuracy will be higher.

## 4. Discussion

Based on traditional SSD and ResNet, this paper proposes an improved detection algorithm R-SSD for human lower body detection, which utilizes ResNet50 instead of VGG16 to improve the feature extraction level of the model. Through Figure 8, the original SSD has a small degradation phenomenon in the late training period, which may be caused by the lack of training data complexity. The SSD based on ResNet avoids this situation. This proves the effectiveness of our method in replacing the backbone network framework (from VGG to ResNet). The experimental results show that the model R-SSD detection accuracy after training reaches 85.1% mAP. Compared with the original SSD, the detection accuracy is improved by 7% mAP. The detection confidence in practical application reaches more than 99% with the enhancement of the object data sample; the proposed method has an obvious effect. Through many experiments, as long as the target object is clearly visible, the improved algorithm can maintain good effectiveness and applicability in industrial or other complex environments.

## 5. Conclusions

This study provides a new and feasible idea to achieve automatic tracking and the method is improved positioning accuracy. This study presents an improved object detection algorithm called R-SSD, which is based on SSD, ResNet, high input resolution and multi-scale feature maps. Compared with the original SSD, the recognition accuracy of R-SSD is improved by 7% mAP for the lower body of humans in AGV. In terms of training data, the strategy of strengthening the object data makes the detection accuracy significantly improved. However, in the next work, we will train the public dataset using our method. For different scenarios, the corresponding data filling can achieve stable and reliable practical application.

## Figures and Tables

**Table 1:** Object multiscale detection network parameters.

Feature Layer Group	Feature Map Size	Default Boxes	
		Distribution	Number
Bottleneck3_4	56 × 56	5	15,680
Bottleneck4_6	28 × 28	5	3920
Bottleneck5_3	14 × 14	5	980
Bottleneck6	7 × 7	5	245
Conv7	4 × 4	5	80
Conv9	1 × 1	5	5

**Table 2:** Relative size of feature detect maps.

Number	Relative Size	Aspect Ratio
1	(0.134, 0.317)	0.42
2	(0.082, 0.183)	0.45
3	(0.395, 0.861)	0.46
4	(0.243, 0.406)	0. 6
5	(0.416, 0.416)	1.0

**Table 3:** Number of labeled images for training.

Number of Images	Number of Lower Body with Complex Images	Number of Lower Body with Simple Images	Total Number of Lower Body
1132	1367	213	1580

**Table 4:** Detection accuracy assessment for SSD and R-SSD on test set.

Method	Input	Data	Pre-Train	BN	Clusters	mAP
SSD	300	Complex	√	×	×	78.1%
R-SSD	300	Complex	√	√	×	80.7%
R-SSD	448	Complex	√	√	×	83.0%
R-SSD	448	Complex + Simple	√	√	×	84.5%
R-SSD	448	Complex + Simple	√	√	√	85.1%
